# CREB1 and ATF1 Negatively Regulate Glutathione Biosynthesis Sensitizing Cells to Oxidative Stress

**DOI:** 10.3389/fcell.2021.698264

**Published:** 2021-06-10

**Authors:** Lina Zhao, Wenjun Xia, Peng Jiang

**Affiliations:** School of Life Sciences, Tsinghua University, Beijing, China

**Keywords:** CREB1, ATF1, GSH, ROS, survival, proliferation

## Abstract

The cAMP response element binding protein (CREB) family activating transcription factor 1 (ATF1) and cAMP response element binding protein 1 (CREB1) have been reported in a diverse group of tumors, however, the mechanistic basis for this remains unclear. Here we found that CREB1 and ATF1 unexpectedly regulate glutathione (GSH) biosynthesis by suppressing the expression of glutamate-cysteine ligase modifier subunit (GCLM) and glutathione synthase (GSS), two key enzymes of GSH biosynthesis pathway. Mechanistic studies reveal that GCLM and GSS are direct transcriptional targets of CREB1 and ATF1. Through repressing the expression of these two enzymes, CREB1 and ATF1 reduce the GSH biosynthesis and the capability of cells to detoxicate reactive oxygen species (ROS), thereby increasing cellular susceptibility to oxidative stress. Therefore, our findings link CREB1 family to cellular metabolism, and uncover a potential therapeutic approach by targeting GCLM or oxidative stress for the treatment of tumors with relatively high expression of CREB1 family proteins.

## Introduction

Reactive oxygen species (ROS) is an important determinant of cancer cells metabolism phenotypes (Rodic and Vincent, 2018). The outcomes of ROS performance in tumor cells are dose-dependent. In general, low level of ROS facilities proliferation, growth, invasion and metastasis of tumor cells, while relatively high level of ROS leads to cell cycle arrest and even cell death. Therefore, it is essential for cancer cells to optimize cellular ROS level to maintain tumor progression (Ahmad et al., 2005; Aykin-Burns et al., 2009; Rodic and Vincent, 2018). To achieve this, tumors cells could increase their antioxidative capacity by upregulating relevant metabolic molecules and the expression of certain antioxidant enzymes. Glutathione (GSH) is thought to be a main small thiol-antioxidant derivative (Veeravalli et al., 2011; Quintana-Cabrera et al., 2012), consisting of L-glutamine, L-cysteine, and L-glycine, found in all mammalian tissues (Lu, 2009). GSH is generally synthesized exclusively in the cytoplasm in two consistently steps at the expenditure of ATP, the first step, which is also a rate-limiting step, catalyzes the generation of γ-glutamylcysteine by the GCL, and the glutathione synthase (GSS) catalyzes the second step to produce GSH. The GCL holoenzyme consists of two separate proteins, a catalytic subunit (GCLC) and a modifier subunit (GCLM) (Krejsa et al., 2010), which are encoded by different genes in humans. GCLC exhibits catalytic activity (Seelig et al., 1984; Lu, 2009) and GCLM is enzymatically inactive but plays an important regulatory role by lowering the Km of GCL for glutamate and raising the Ki for GSH (Huang et al., 1993a,b). GSH synthesis pathway is severely activated by some stimuli including oxidative stress.

cAMP response element binding protein 1 (CREB1) belongs to the bZIP transcription factors. The other two members of CREB1 family are activating transcription factor 1 (ATF1) and cAMP response element modulator (CREM). CREB1 and ATF1 both express ubiquitously, whereas CREM mainly expresses in neuroendocrine tissues (Mayr and Montminy, 2001). Although CREB1 has been implicated in the physiology of nerve and most recently in some cancer cells by cooperating with some protein kinases or small molecules, including PKA, PKB (AKT), MAPK, cAMP, and Ca^2+^ to inhibit apoptosis and promote cell survival in unstressed situation (Bonni and Brunet, 1999; Shankar et al., 2005; Chhabra et al., 2007; Pigazzi et al., 2007; Aggarwal et al., 2008; Shukla et al., 2009), whether CREB families play a role in modulating tumor cellular metabolism remains unknown.

## Results

**FIGURE 1:**
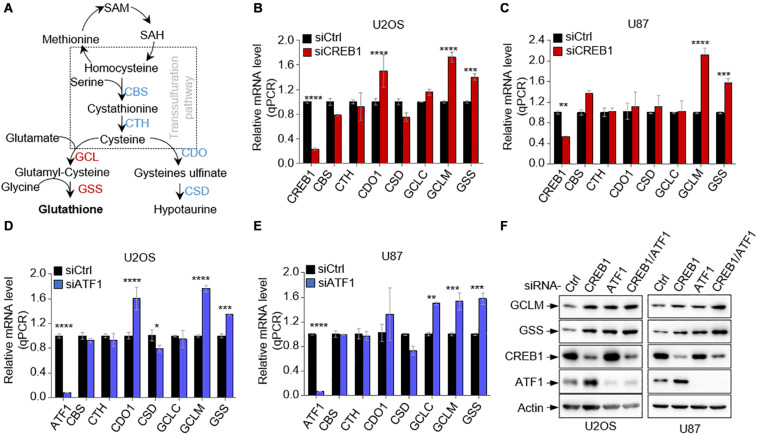
CREB1 and ATF1 repress the expression of GCLM and GSS. **(A)** Schematic depicting the GSH synthesis pathway and its associated hypotaurine synthesis pathway. **(B,C)** Relative mRNA levels of enzymes of GSH synthesis pathway in U2OS cells **(B)** and U87 cells **(C)** transfected with CREB1 siRNA or control siRNA. **(D,E)** Relative mRNA levels of GSH synthesis enzymes in U2OS cells **(D)** and U87 cells **(E)** transfected with control siRNA or ATF1 siRNA as indicated. **(F)** U2OS and U87 cells expressing control siRNA, or siRNAs against CREB1, ATF1, or both (CREB1/ATF1) as indicated. Protein expression was analyzed by Western blot. Data are means ± SD. (*n* = 3), *p < 0.05, **p < 0.01, ***p < 0.001, ****p < 0.00001.

To determine if CREB1 regulates GSH synthesis, we examined the effect of CREB1 silencing on the expression of several major enzymes of the GSH synthetic pathways in multiple cell lines including U2OS and U87 cells (Figure 1A). Interestingly, while the mRNA level of most enzymes remained approximately the same (CTH and GCLC) or changed inconsistently (CBS, CDO1, and CSD), the mRNA levels of GCLM and GSS increased consistently and significantly when CREB1 was knocked down in U2OS, U87, and HepG2 cells (Figures 1B,C and Supplementary Figure 1A). Similar findings were found in A549, H1299, and H1975 cells. CREB1 ablation led to an increase in the expression of GCLM and GSS expression (Supplementary Figures 1B–D). Like CREB1, knocking down of ATF1 expression in U2OS and U87 cells resulted in strong increase in mRNA levels of both GCLM and GSS (Figures 1D,E). Moreover, upon ATF1 knockdown, GCLM and GSS mRNA levels augmented in multiple cell lines including HepG2, A549, H1299, and H1975 cells (Supplementary Figures 1E–H). Results from western blot analysis revealed that silencing of either of CREB1, ATF1 or both enhanced the protein levels of GCLM and GSS in U2OS and U87 cells, as well as in HepG2 and A549 cells (Figure 1F and Supplementary Figures 1I,J). Similarly, sgRNA-mediated knockout of CREB1 and ATF1 in U2OS cells strongly boosted the expression of GCLM and GSS (Supplementary Figure 1K). These findings were further confirmed by overexpression of CREB1 and ATF1. In line with the knockdown data, enforced expression of CREB1 and ATF1 led to strong reduction in protein levels of GCLM and GSS in U2OS cells (Supplementary Figure 1L). Collectively, these findings show that both CREB1 and ATF1 negatively regulate the expression of GCLM and GSS, two key enzymatic steps of GSH synthesis pathway.

**FIGURE 2:**
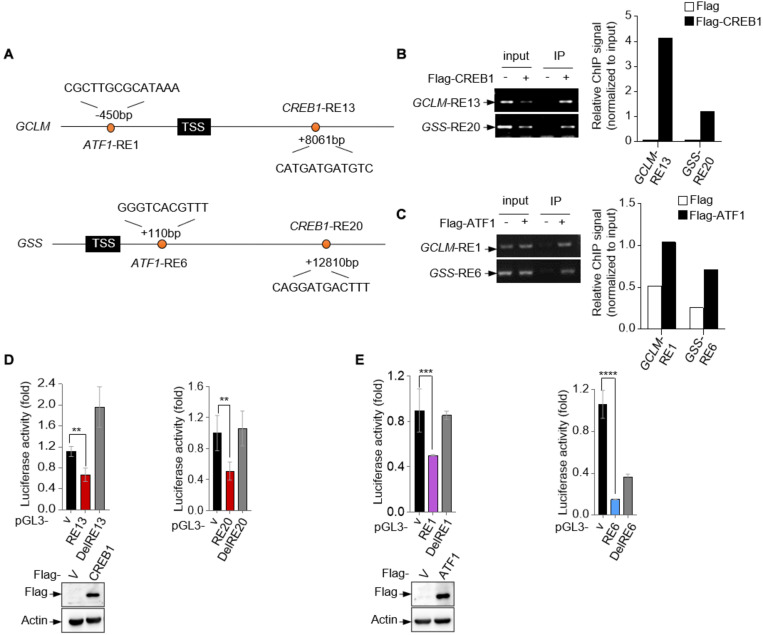
GCLM and GSS are transcriptional targets of CREB1 and ATF1. **(A)** Schematic representation of human *GCLM* and *GSS* genomic structure. The sequences of potential CREB1 and ATF1 response elements *GCLM*-RE13, *GSS*-RE20, *GCLM*-RE1, and *GSS*-RE6 are shown. **(B,C)** 293T cells were transfected with Flag-CREB1 **(B)**, Flag-ATF1 **(C)** or vector control plasmid. Cell lysates were used for ChIP assay using anti-Flag antibody. Bound DNA was amplified by PCR. Results are representative of three independent experiments. **(D,E)** Luciferase constructs containing indicated responsive elements (REs) were transfected into 293T cells together with Flag-CREB1 **(D)**, Flag-ATF1 **(E)** and vector control, respectively. Renilla vector pRL-CMV was used as a transfection internal control. The relative luciferase activity was normalized to the co-transfected Renilla activity. Data are means ± SD. (*n* = 3), **p < 0.01, ***p < 0.001, ****p < 0.0001.

To investigate whether CREB1 and ATF1 are the transcription factors for GCLM and GSS, we analyzed human *GCLM* and *GSS* gene sequences for potential response elements of CREB family proteins, which share the conservative sequence of 5′-TGACGTCA-3′. Potentially, we identified a response element (RE) for CREB1 and ATF1, respectively, in each of them (Figure 2A). Chromatin immunoprecipitation (ChIP) assays showed that CREB1 and ATF1 could bind to these REs (Figures 2B,C). Next, we cloned the fragments of *GCLM* and *GSS* genes containing the corresponding response elements into the promotor region of a luciferase reporter plasmid, and found that CREB1 and ATF1 repressed the luciferase activity driven by these REs, but not the mutant ones (Figures 2D,E). These results together indicate that GCLM and GSS may be the transcriptional targets for CREB1 and ATF1.

**FIGURE 3:**
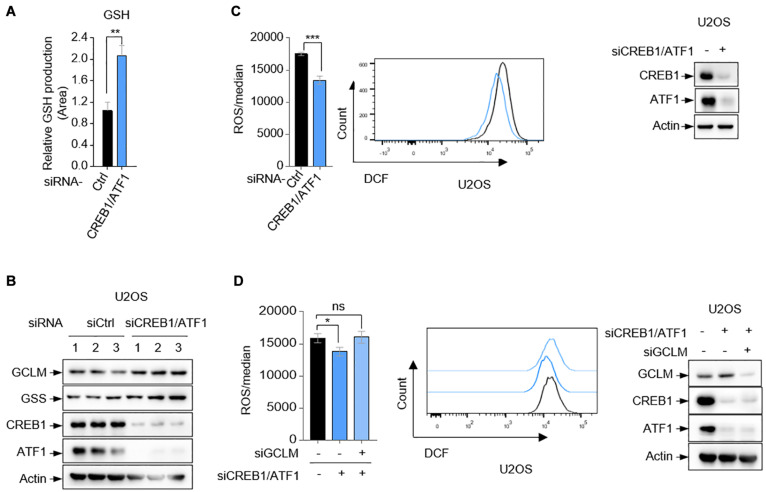
CREB1 and ATF1 inhibit intracellular ROS. **(A,B)** U2OS cells transfected with control siRNA, or CREB1 siRNA together with ATF1 siRNA (siCREB1/ATF1) were assayed for intracellular GSH production by LC-MS. **(C)** Relative ROS level in U2OS cells transfected with CREB1 and ATF1 siRNAs (siCREB1/ATF1), or control siRNA was determined by flow cytometry analysis. Protein expression was analyzed by Western blot. **(D)** U2OS cells were transfected with control, CREB1, ATF1 and/or GCLM siRNA as indicated. Relative ROS level was analyzed by flow cytometry. Protein expression was analyzed by Western blot. Data are means ± SD. (*n* = 3), *p < 0.05, **p < 0.01, ***p < 0.001.

GCLM and GSS are essential for GSH synthesis (Li et al., 2016). Using small interfering RNA (siRNA) to simultaneously knock down the expression of CREB1 and ATF1, we observed that GSH production was substantially increased in U2OS cells (Figures 3A,B). GSH is considered to be one of the most important scavengers of reactive oxygen species (ROS). Consistent with this, ablation of CREB1 and ATF1 decreased the cellular level of ROS in both U2OS, U87, and HepG2 cells as determined by DCF staining (Figure 3C and Supplementary Figures 2A,B). The specificity of the assay was shown by an increase in the detected ROS upon G6PD silencing (Supplementary Figure 2C). G6PD is the first and rate-limiting enzyme of the PPP pathway, which is the main source of intracellular antioxygen NADPH (nicotinamide adenine dinucleotide phosphate, reduced) (Jiang et al., 2011). Conversely, overexpression of CREB1 and ATF1 led to increased intracellular ROS in U2OS cells (Supplementary Figure 2D). Consistent with previous study (Huang et al., 1993b; Chhabra et al., 2007), GSS depletion showed no effect on ROS level, which could be due to the complex GSH-independent action(s) of GSS (Supplementary Figure 2E). Nevertheless, silencing GCLM resulted in an increase in ROS level (Supplementary Figure 2E). Moreover, knockdown of GCLM alone elevated intracellular level of ROS, and reversed the suppressive effect of depletion of CREB1 and ATF1 on ROS in U2OS cells (Figure 3D). Collectively, these results indicate that CREB1 and ATF1 have a role in maintaining ROS though downregulation of GCLM and GSS expression.

**FIGURE 4:**
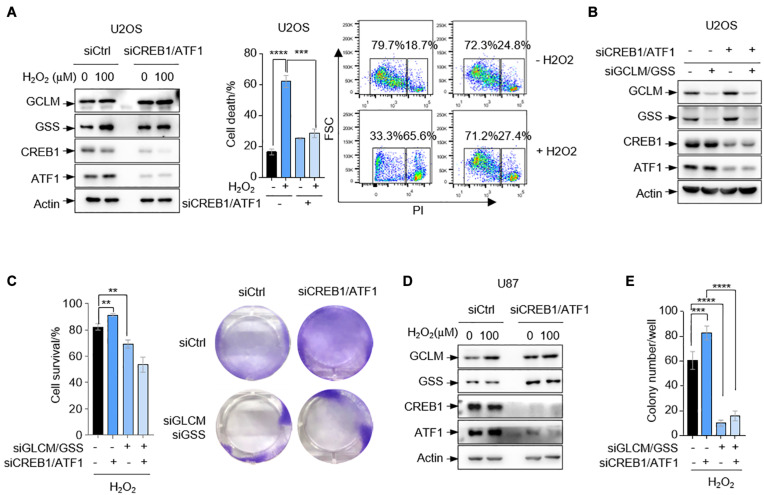
CREB1 and ATF1 inhibit cell survival under oxidative stress. **(A)** U2OS cells transfected with control, or CREB1 and ATF1 siRNAs (siCREB1/ATF1) were treated with 100 μM H_2_O_2_ for 24 h. Protein expression was measured by Western blot analysis. Cell death was analyzed by flow cytometry after PI staining. **(B,C)** U2OS cells treated with control, CREB1, ATF1, GCLM, and/or GSS siRNAs as indicated. Protein expression was analyzed by Western blot **(B)**, and cell survival was assayed by crystal violet staining **(C)**. **(D)** U87 cells transfected with control siRNA, or siRNAs against CREB1 and ATF1 as indicated were treated with 100 μM H_2_O_2_ for 24 h. Protein expression was analyzed by Western blot. **(E)** U87 cells were transfected with control, CREB1, ATF1, GCLM, and/or GSS siRNAs for colony formation assay. Numbers of colonies with a diameter greater than 20 μm were quantified. Data are means ± SD. (*n* = 3), **p < 0.01, ***p < 0.001, ****p < 0.0001.

The above findings that CREB1 and ATF1 unexpectedly reduce cell capability to scavenge ROS promoted us to investigate whether cells with ablation of CREB1 and ATF1 are resistant to oxidative stress. Indeed, deletion of CREB1 and ATF1 in U2OS cells resulted in a reduction in cell death as determined by FACS analysis when cells were cultured in medium containing 100 μM H_2_O_2_ (Figure 4A). To examine whether GCLM and GSS are involved in CREB1- and ATF1-mediated cell death under oxidative stress, we knocked down the expression of GCLM and GSS in U2OS cells expressing siRNAs against CREB1 and ATF1. Again, depletion of CREB1 and ATF1 led to increased cells viability, and this effect was reversed by GCLM and GSS silencing in the presence of 100 μM H_2_O_2_ (Figures 4B,C). We next confirmed these findings by an anchorage-independent growth in soft agar, an *in vitro* measurement of tumorigenicity. Depletion of CREB1 and ATF1 caused increased capacity of U87 cells to form colonies in soft agar supplied with 100 μM H_2_O_2_, and knockdown of GCLM and GSS almost totally reduced the anchorage-independent growth regardless of CREB1 and ATF1 status (Figures 4D,E and Supplementary Figure 3).

## Conclusion

In this work, we found a previously unappreciated role for CREB family proteins in controlling GSH synthesis. Through downregulation of the expression of GCLM and GSS, CREB1 and ATF1 unexpectedly reduce GSH generation and thereby dampen the cellular ability to scavenge ROS, leading to increased susceptibility of cells to oxidative stress. Intriguingly, GSS seems not to be essential for ROS detoxification, but still acts a key metabolic enzyme of the GSH *de novo* synthetic pathway. To elucidate the underlying mechanism(s) for these unexpected observations would be of great interest. One potential expectation is that GSS possess ROS-related but GSH synthesis-independent functions, which could comprise the effect of GSH production on ROS. Nevertheless, GCLM downregulation appears to be sufficient to abolish ROS reduction in cells depleted of CREB family proteins CREB1 and ATF1. CREB1 has been implicated in supporting tumor cell proliferation and survival in some types of tumor cells, indicating that CREB1 might be an oncogenic protein that can help tumor cell to proliferate in certain situations (Thway and Fisher, 2012). Here our findings reveal a metabolic mechanism for CREB1-mediated tumor cell survival, and also suggest that targeting GCLM or oxidative stress potentially could be a therapeutic approach for treatment of tumors with relatively high expression of CREB1 family proteins.

## Materials and Methods

### Antibodies and Reagents

Antibodies against the following proteins/epitopes were used for immunoblot with the sources, catalog numbers, and dilutions indicated: Actin (Easy Bio, BE0037-10, 1:5000), Flag (Sigma-Aldrich, F3165, 1:5000), GCLM (Proteintech, 14241-1-AP, 1:1000), GSS (Santa Cruz Biotechnology, sc-166882, 1:1000), CREB1 (Proteintech, 12208-1-AP, 1:1000), and ATF1 (Proteintech, 11946-1-AP, 1:1000).

The ANNEXIN V-FITC/PI Apoptosis Detection Kit were purchased from Solarbio. The following regents were purchased from Sigma-Aldrich: crystal violet (CV), 0.4% trypan blue solution, 2′,7′-dichlorofluorescin diacetate (DCF), propidium iodide (PI), puromycin.

### Cell Culture

Cells were maintained in standard culture conditions without any antibiotic. A549, H1299, HEPG2, H1975, and U87 were from ATCC (Manassas, VA, United States). All cells were cultured in a 5% CO_2_ humidified incubator (Thermo Fisher Scientific, United States) at 37°C. A549 cell line was maintained in standard Dulbecco’s modified Eagle’s medium (DMEM) (Thermo Fisher Scientific, C11995500BT) with 10% fetal bovine serum (FBS) (GEMINI, 100–106). U2OS cells were cultured in McCoy’s 5 A Medium, U87 and HEPG2 cells in MEM supplemented with non-essential amino acid (Gibco,11140050). H1299 and H1975 cells were cultured in standard RPMI1640 medium (Thermo Fisher Scientific, 11875093) with 10% FBS. Unless indicated otherwise, all cells were cultured without the addition of penicillin-streptomycin and for no more than two consecutive months, and were routinely examined for mycoplasma contamination.

### Genes Knock Down With siRNA

The flowing siRNAs were purchased from GenePharma. The sequences were: CREB1#1, 5′-AAA CAUUAACCAUGACCAATT-3′; CREB1#2, 5′-GGUGCCAA CUCCAAUUUACTT-3′; ATF1#1, 5′-AGGUACAACUAUUC UUCAGUA-3′; ATF1#2, 5′-GCUCAACAGGUAUCAUCUUUA-3′; GCLM; 5’-GCAAGUUUCCAAGAAGCUCTT-3′; GSS, 5′- CCAUCAAACAGGAUGACUUUATT -3′ G6PD, 5′-ACGAGCUGAUGAAGAGAGUGGGUUU-3′. siRNAs were transfected into cells using Lipo2000 Transfection Agent (Thermo Fisher Scientific, 12566014) following the manufacturer’s instruction.

### Plasmids

The coding sequences corresponding to the full-length human CREB1 and ATF1 were amplified by polymerase chain reaction (PCR) from cDNA library of 293T cells and then cloned into Prk5-flag empty vector as indicated. The cloning sequences are as follows: CREB1, 5′-CGCGGATCCGCGATGACCATGGAATCTGGAGCCG-3′ (forward) and 5′-CCCAAGCTTGGGTTAATCTGAT TTGTGGCAGTAAAGGTCCT-3′ (reverse); ATF1, 5′-TGC TCTAGAATGGAAGATTCCCACAAGAGTAC-3′ (forward) and 5′-CCCAAGCTTTCAAACACTTTTATTGGAATAAAGAT CC-3′ (reverse). sgRNAs were made in px330-GFP vector. The sequences were: CREB1:5′-CACCGGGGCAGACAGTTC AAGTCCA-3′(sense) and 5′-AAACTGGACTTGAACTGT CTGCCCC-3′ (antisense); ATF1: 5′-CACCGGAACGCTG ACGTCATCAACCCG-3′ (sense) and 5′-AAACGCACCGAC TCGGTGCCAC-3′ (antisense).

### Semi-Quantitative RT-PCR and Quantitative RT-PCR

RNA was extracted by TRIzol (15596-018, Invitrogen) according to the manufacturer’s instruction. 2 μg RNA of each sample was reversed to complementary DNA by First-strand cDNA Synthesis System (Thermo Fisher Scientific, catalog No. K1622), and 0.2 μg cDNA was used as a template to perform PCR. Quantitative PCR were performed on CFX96 Real-Time PCR System (Bio-Rad, United States) and the amplifications were done using the SYBR Green PCR Master Mix (Gene Star, China). The primer pairs were: CREB1, 5′-ATTCACAGGAGTCAGTGGATAGT-3′ (forward) and 5′-CACCGTTACAGTGGTGATGG-3′ (reverse); ATF1, 5′-AGGACTCATCCGACAGCATAG-3′ (forward) and 5′-TTCTGCCCCGTGTATCTTCAG-3′ (reverse); CBS, 5′-GTCA GACCAAGTTGGCAAAGT-3′ (forward) and 5′-CACCCCGA ACACCATCTGC-3′ (reverse); CTH, 5′-AAAGACGCCTCCT CACAAGG-3′ (forward) and 5′-AAGGCAATTCCTAGTG GGATTTC-3′ (reverse); CDO1, 5′-TCCATTGGCTTACATCG AGTAGA-3′ (forward) and 5′-CCCGAAGTTGCATTTGGAGT-3′ (reverse); CSD, 5′-AGAAGCGGGAAGGGTTTGAG-3′ (forward) and 5′-CCTTTCGTGGTAATCTGGACTC-3′ (re verse); GCLC, 5′-GGAGACCAGAGTATGGGAGTT-3′ (fo rward) and 5′-CCGGCGTTTTCGCATGTTG-3′ (reverse); GCLM, 5′-CATTTACAGCCTTACTGGGAGG-3′ (forward) and 5′-ATGCAGTCAAATCTGGTGGCA-3′ (reverse); GSS, 5′-GGGAGCCTCTTGCAGGATAAA-3′ (forward) and 5′-GAATGGGGCATAGCTCACCAC-3′ (reverse); β-actin, 5′GAC CTGACTGACTACCTCATGAAGAT-3′ (forward) and 5′-GTCACACTTCATGATGGAGTTGAAGG-3′ (reverse). The fold changes of gene expression are normalized to β-actin.

### Western Blot Analysis

Whole-cell lysates were obtained using modified RIPA lysis buffer (10 mM Tris–HCl at pH 7.5, 5 mM EDTA, 150 mM NaCl, 1% NP-40, 1% sodium deoxycholate, 0.025% SDS, and added protease cocktail freshly). Cells were washed and incubated in lysis buffer for 30 min on ice, and boiled in 5x loading buffer. Protein samples were resolved by SDS-PAGE and transferred onto nitrocellulose membrane. The membrane was then blocked in 5% skimmed milk in TBST and probed with the indicated antibodies.

### Chromatin Immunoprecipitation (ChIP) and Reporter Assays

We used JASPAR^1^ to identify the potential CREB1 and ATF1 response elements in *GCLM* and *GSS* genes. The ChIP analysis was performed with some modifications. The response elements were amplified by PCR. The primer pairs were: *GCLM* RE1, 5′-CCGCTCGAGGTCCTTGAAGGTAGCAAAGCGA-3′ (forward) and 5′-CCCAAGCTTCGGCCGCCCCAGG GTGGGCTTC-3′ (reverse); *GCLM* RE13, 5′-GAGTCAGAAT AGAACTTCAACTT-3′ (forward) and 5′-TCTCTTCATG TAACTTGCCCAG-3′ (reverse); *GSS* RE6, 5′-CCCT CGAGTCTGCCCCGCCTGCTCTTTAA-3′ (forward) and 5′-CCCAAGCTTCTAGACACTAGTTGCCCCGCTC-3′ (reverse); *GSS* RE20, 5′-GATTCAGGAGTCAATGACTGA-3′ (forward) and 5′-GACAGGTTACAGACTGGAGTAG-3′ (reverse).

For reporter assay, the sequences for CREB1 response elements in *GCLM* and *GSS* gene are: *GCLM*-RE13, 5′-CATGATGATGTC-3′, *GSS*-RE20, 5′-CAGGATGACTTT-3′. The sequences for ATF1 response elements in *GCLM* and *GSS* gene are: *GCLM*-RE1, 5′-CCGCTTGCGCATAAA-3′; *GSS*-RE6, 5′-GGGTCACGTTT-3′. These binding regions were cloned into pGL3-basic vector (Promega, Madison, WI, United States, catalog No: E1751). The luciferase activity was determined using a Dual Luciferase Assay System (Promega, catalog No: E1910). Transfection efficiency was normalized on the basis of the Renilla luciferase activity.

### Measurements of GSH and ROS

Glutathione was determined using liquid chromatography-mass spectrometry (LC-MS). We used cold 80% methanol to extract metabolites analyzed by LC-MS. The measured mass isotopomer distributions were corrected by natural abundances.

Reactive oxygen species levels were determined as followed. Cells were incubated at 37°C for 30 min in PBS containing 10 μM 2′,7′-dichlorodihydrofluorescein diacetate (H2-DCFDA, Sigma). Afterward, the cells were washed twice in PBS, treated with trypsin, and resuspended in PBS. Fluorescence was immediately measured using a FACS Flow Cytometer (BD LSRFortessa SORP).

### Cell Survival Assay

Cells were transfected with siRNA for 24 h then seeded in 6-well plates in triplicates at the density of 10^^5^ cells per well in 2 ml medium supplemented with 10% FBS. Cells were added with 100 μM H_2_O_2_ after fixing on the plate, then incubated for 24 h. For trypan blue staining, cells were harvested with trypsin, washed once and resuspended in PBS, then added 10 μl trypan blue into cell suspension at volume ratio 1:1. Live cell ratio was determined by counting using a hemocytometer. For CV staining, cells were fixed with 10% formalin for 5 min and stained with 0.05% CV for 30 min. After washed with distilled water, cells were photographed.

### Soft Agar and Colony Formation Assay

U87 cells were transfected with Luciferase siRNA or CREB1 and ATF1 siRNA for 24 h and then suspended in 1 ml MEM medium supplemented with or without 100 μM H_2_O_2_ plus 20% FBS containing 0.3% agarose and plated on the firm 0.6% agarose base in the 12-well plates (2,000 cells per well). Cells were then cultured in a 37°C with 5% CO_2_ incubator. After 2–3 weeks, colonies were stained with 0.05% crystal violet in PBS for 1 h. Counted under a microscope and photoed after colonies turning into blue.

### Statistical Analysis

All experiments in this work were performed at least three times independently. Multiple groups comparison was analyzed by two-way ANOVA with multiple comparison test by GraphPad Prism 7. For experiments with only two groups, a two-tailed Student’s *t*-test was used to calculate the *P*-value. *P* < 0.05 was considered significant. ^∗^*p* < 0.05, ^∗∗^*p* < 0.01, ^∗∗∗^*p* < 0.001, ^****^p < 0.0001, NS, not significant.

## Data Availability Statement

The raw data supporting the conclusions of this article will be made available by the authors, without undue reservation.

## Author Contributions

LZ, WX, and PJ designed the experiments. LZ and WX performed all the experiments. LZ and PJ wrote the manuscript. All authors reviewed and commented on the manuscript.

## Conflict of Interest

The authors declare that the research was conducted in the absence of any commercial or financial relationships that could be construed as a potential conflict of interest.

## Footnotes

1 http://jaspar.genereg.net
