# Supplementary material for: CREB1 and ATF1 Negatively Regulate Glutathione Biosynthesis Sensitizing Cells to Oxidative Stress
Source: Front Cell Dev Biol. 2021 Jun 10;9:698264. doi: 10.3389/fcell.2021.698264 (PMC8223876; doi:10.3389/fcell.2021.698264)
Supplement: Supplementary file 1 [file Data_Sheet_1.PDF]

Figure S1

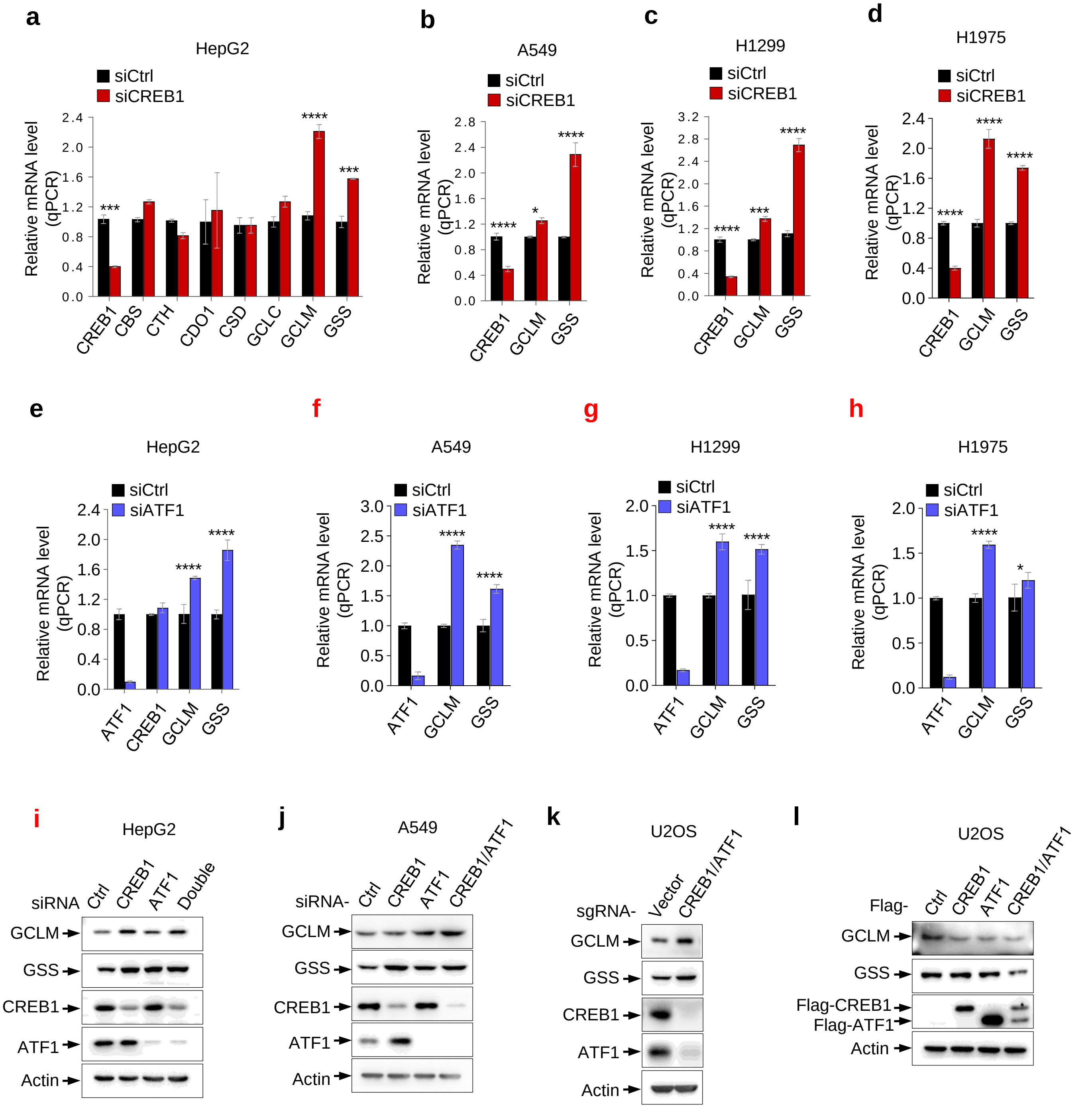
a
HepG2
siCtrl
siCREB1
Relative mRNA level (qPCR)
CREB1
CBS
CTH
CDO1
CSD
GCLC
GCLM
GSS
b
A549
c
H1299
d
H1975
e
HepG2
siATF1
ATF1
f
A549
g
H1299
h
H1975
i
HepG2
siRNA
Ctrl
ATF1
Double
Actin
j
A549
siRNA-
CREB1/ATF1
k
U2OS
sgRNA-
Vector
l
U2OS
Flag-
Flag-CREB1
Flag-ATF1

Supplementary Fig. 1. CREB1 and ATF1 represses the expression of GCLM and GSS.

a, Relative mRNA levels of GSH synthesis enzymes in HepG2 cells transfected with control or CREB1 siRNA.

b-d, Relative mRNA levels of GCLM and GSS in A549 cells (**b**), H1299 cells (**c**), and H1975 cells (**d**) transfected with control or CREB1 siRNA. Results are representative of three independent experiments.

e-h, Relative mRNA levels of GCLM and GSS in HepG2 cells (**e**), A549 cells(f), H1299 cells (**g**), and H1975 cells (**h**) transfected with control or ATF1 siRNA. Results are representative of three independent experiments.

i-j, Lysates from HepG2 cells, A549 cells transfected with control, CREB1 and/or ATF1 siRNAs were analyzed by Western blot.

k, Lysates from U2OS cells stably transfected with px330-vector, or px330-sgCREB1 together with px330-sgATF1 were analyzed by Western blot.

l, Lysates from U2OS cells transfected with Flag-vector, Flag-CREB1 and/or Flag-ATF1 plasmids were analyzed by Western blot.

Data are means ± S.D. (n = 3). *p<0.05, **p<0.01, ***p<0.001.

Figure S2

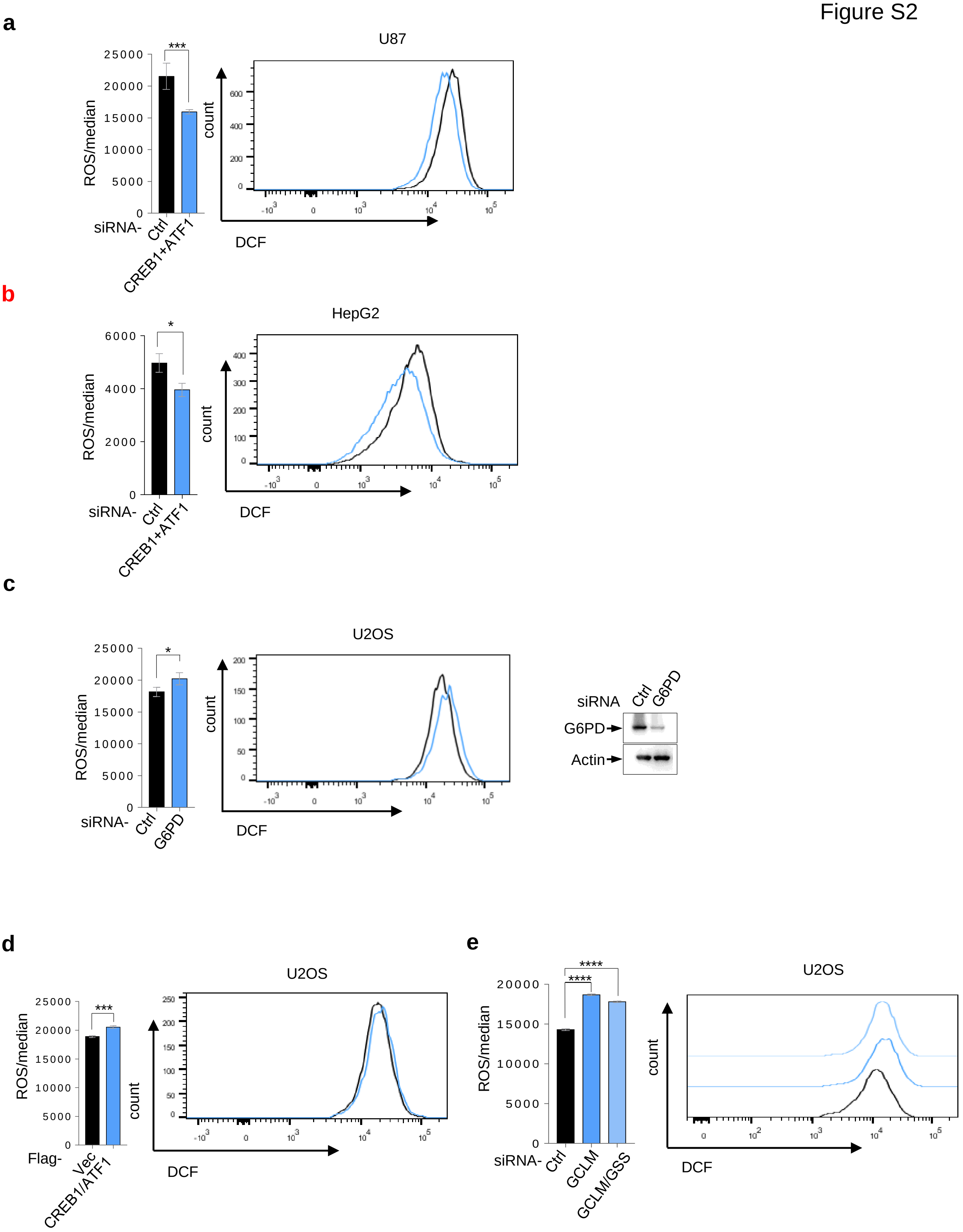
a
U87
ROS/median
25000
20000
15000
10000
5000
0
***
siRNA-
Ctrl
CREB1+ATF1
count
DCF
b
HepG2
6000
4000
2000
*
c
U2OS
G6PD
siRNA
G6PD
Actin
d
Flag-
Vec
CREB1/ATF1
e
****
GCLM
GCLM/GSS

Supplementary Fig. S2. CREB1 and ATF1 inhibit intracellular ROS.

a, Relative ROS level in U87 cells transfected with control, or CREB1 and ATF1 siRNAs was determined by flow cytometry analysis.

b, Relative ROS level in HepG2 cells transfected with control siRNA, or siRNAs against CREB1 and ATF1 was determined by flow cytometry analysis.

c, Relative ROS level in U2OS cells transfected with control or G6PD siRNAs was determined by flow cytometry analysis.

d, Relative ROS level in U2OS cells transfected with Flag-vector, Flag-CREB1 together with Flag-ATF1 was determined by flow cytometry analysis.

e, Relative ROS level in U2OS cells transfected with control, GCLM, GCLM and/or GSS siRNAs as indicated was determined by flow cytometry analysis.

All data are repeated at least three times.

Figure S3

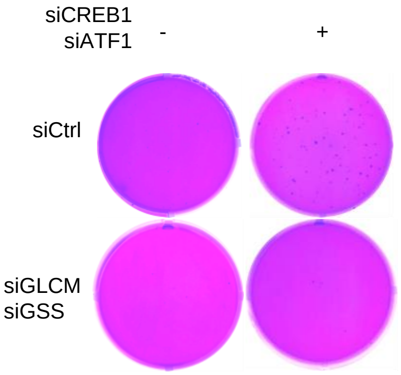
siCREB1
siATF1
-
+
siCtrl
siGLCM
siGSS

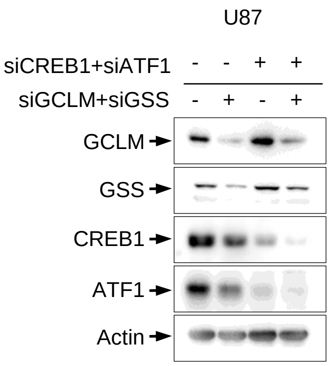
U87
siCREB1+siATF1
-
-
+
+
siGCLM+siGSS
-
+
-
+
GCLM
GSS
CREB1
ATF1
Actin

Supplementary Fig. 3. CREB1 and ATF1 suppresses colonies formation under oxidative stress condition. U87 cells were transfected with control, CREB1, ATF1 and/or GCLM siRNAs as indicated. Colonies of U87 were visualized by crystal violet staining. Protein expression was determined by Western blot. Data are representative of three independent experiments.
